# Taurine Supplementation to Plant-Based Diets Improves Lipid Metabolism in Senegalese Sole

**DOI:** 10.3390/ani13091501

**Published:** 2023-04-28

**Authors:** Cláudia Aragão, Rita Teodósio, Rita Colen, Nadège Richard, Ivar Rønnestad, Jorge Dias, Luís E. C. Conceição, Laura Ribeiro

**Affiliations:** 1 Centre of Marine Sciences (CCMAR), 8005-139 Faro, Portugal; rteodosio@ualg.pt (R.T.); rcolen@ualg.pt (R.C.); 2 Universidade do Algarve, 8005-139 Faro, Portugal; 3 Phileo by Lesaffre, 59700 Marcq-en-Baroeul, France; n.richard@phileo.lesaffre.com; 4 Department of Biological Sciences, University of Bergen, 5020 Bergen, Norway; ivar.ronnestad@uib.no; 5 SPAROS Lda., 8700-221 Olhão, Portugal; jorgedias@sparos.pt (J.D.); luisconceicao@sparos.pt (L.E.C.C.); 6 IPMA—Portuguese Institute for the Ocean and Atmosphere/EPPO—Aquaculture Research Station, 8700-194 Olhão, Portugal; lribeiro@ipma.pt

**Keywords:** aquaculture, *Solea senegalensis*, flatfish, fishmeal replacement, taurine, bile acids, lipid utilisation, metabolic trials

## Abstract

**Simple Summary:**

In contemporary dietary formulations for aquaculture, the amounts of fishmeal are being constantly reduced, and the inclusion of plant-protein sources is now a standard procedure. This approach may result in an unbalanced supply of selected nutrients, from which taurine was identified. Taurine is an amino acid that plays important physiological roles and is included in bile salts, which are essential for the emulsion, digestion, and absorption of dietary lipids and fat-soluble vitamins. In mammals, a hypolipidemic effect of taurine has been described. Senegalese sole (*Solea senegalensis*) is a marine fish species with increasing importance for aquaculture production in Southern European countries, with a high market value and low tolerance to dietary lipid levels. Thus, this study tested the effects of taurine supplementation to low-fishmeal diets on the physiological responses of Senegalese sole, with an emphasis on lipid metabolism. The results show that dietary inclusion of high levels of plant-protein sources to replace marine ingredients resulted in negative effects on lipid metabolism, due to the resultant low bile-acid concentration and/or the limited availability of taurine for bile-salt emulsification. Taurine supplementation mitigated part of the negative effects of plant-based diets, leading to better lipid utilisation.

**Abstract:**

Taurine is a sulphur-containing amino acid with important physiological roles and a key compound for the synthesis of bile salts, which are essential for the emulsion and absorption of dietary lipids. This study aimed to evaluate the effects of taurine supplementation to low-fishmeal diets on the metabolism of taurine, bile acids, and lipids of Senegalese sole. A fishmeal (FM) and a plant-protein-based (PP0) diet were formulated, and the latter was supplemented with taurine at 0.5 and 1.5% (diets PP0.5 and PP1.5). Diets were assigned to triplicate tanks containing 35 fish (initial weight ~14 g) for 6 weeks. Fish from the PP0 treatment presented lower taurine and bile-acid concentrations compared with the FM treatment, and a downregulation of *cyp7a1* and *abcb11* was observed. Triolein catabolism decreased in PP0-fed fish, resulting in increased hepatic fat content and plasma triglycerides, while no effects on plasma cholesterol were observed. Taurine supplementation to plant-based diets resulted in a higher taurine accumulation in fish tissues, increased bile-acid concentration, and upregulation of *cyp7a1* and *abcb11*. Hepatic fat content and plasma triglycerides decreased with increasing dietary taurine supplementation. Taurine supplementation mitigated part of the negative effects of plant-based diets, leading to better lipid utilisation.

## 1. Introduction

Aquaculture has grown rapidly during the last few years, fuelled by the rising per capita consumption of seafood [1]. This growth was supported by a significant research effort to increase the industry’s sustainability. During the last two decades, a lot of research was focused on the potential of plant-derived ingredients as an alternative to substitute fishmeal and fish oil in aquafeed formulations [1]. However, the dietary replacement of fishmeal with plant ingredients may result in an unbalanced supply of key nutrients. Among these, taurine (2-aminoethanesulfonic acid) was identified, since it is virtually absent in terrestrial plant ingredients, while it is particularly abundant in the marine food chain [2]. Taurine is an end product of the metabolism of sulphur-containing amino acids. Taurine synthesis occurs in the liver, but it seems to be limited in fish, especially in marine fish, due to the low levels or absence of rate-limiting enzymes in taurine biosynthesis [2,3]. Therefore, taurine is considered a conditionally essential nutrient for marine fish [4], and when feeding these species with terrestrial plant-protein-based diets, taurine requirements may not be fulfilled. Although considered a sulphur amino acid, taurine is not incorporated into proteins, remaining free in the cytosol, and it takes part in many important physiological processes, such as osmotic regulation, membrane stabilisation, antioxidation, eye development, and control of plasma cholesterol levels [2,3]. Taurine is also involved in the synthesis of bile salts, which are essential for the emulsion, digestion, and absorption of dietary lipids and fat-soluble vitamins. Bile acids are synthesised from cholesterol in the liver, with cholesterol 7α-hydroxylase acting as the rate-limiting enzyme [5]. Bile acids are then conjugated with an amino acid, which in fish is mainly taurine, to form bile salts that are secreted into the bile [6]. The secretion of compounds into the bile canaliculi is mediated by several ATP-binding cassette (ABC) transporters expressed at the canalicular membrane of hepatocytes [7]. Considering the key role of taurine in bile-acid synthesis, its importance in lipid metabolism is evident.

Several works studied the effects of dietary taurine supplementation, with contradictory results found across the literature depending on the species, developmental stage, and diet formulation (marine or plant-based). Dietary supplementation to fishmeal-based diets had no effects on the growth performance of several freshwater and marine fish species [8,9,10]. Although it was suggested that taurine-synthesis capacity was more limited in marine than in freshwater fish species [11], some studies showed an improved growth performance in carps (*Mylopharyngodon piceus* and *Ctenopharyngodon idella*), catfishes (*Clarias gariepinus* and *Pelteobagrus fulvidraco*), Nile tilapia (*Oreochromis niloticus*), and rainbow trout (*Oncorhynchus mykiss*) when taurine was included in high- or all-plant diets [8,12,13,14,15,16]. However, even when feeding low-fishmeal diets, no effects on growth performance were found in zebrafish (*Danio rerio*) and Atlantic salmon (*Salmo salar*) [17,18]. In marine fish species, several studies reported an increase in growth performance and/or feed efficiency with an increase in dietary taurine content, especially when using low-fishmeal diets [9,10,19,20,21,22,23,24,25,26].

Even when no effects were observed on growth performance, taurine supplementation may enhance the physiological condition of the fish. Several works report that dietary taurine supplementation to plant-based diets enhanced antioxidant status, immunity, and health in several freshwater and marine fish species [12,14,15,17,21,27,28,29]. To the best of our knowledge, negative effects on liver health were only reported for Persian sturgeon (*Acipenser persicus*) fed high-plant diets supplemented with taurine [30]. However, further studies demonstrated that dietary taurine inclusion levels in that study were supraoptimal [31]. Furthermore, taurine deficiency impaired bile pigment (biliverdin and bilirubin) production and secretion, inducing green liver syndrome in species such as red seabream (*Pagrus major*) and yellowtail (*Seriola quinqueradiata*) [22,23,32].

In mammals, taurine exerts a hypolipidemic effect, with the prevention of increasing levels of cholesterol and triglycerides [33]. As taurine is the only amino acid to conjugate with bile acids in teleost fish [6], it is not surprising that several studies report an increase in conjugated or total bile-acid concentration concomitantly with an increase in dietary taurine content [20,28,34,35,36]. Since bile-acid synthesis results from cholesterol catabolism [5], a decrease in plasma cholesterol levels was correlated with increasing bile-acid synthesis in fish fed low-fishmeal diets supplemented with taurine [13,14,28]. However, in other studies, taurine supplementation to low-fishmeal diets had no effects on cholesterol levels, even if an increased concentration of bile acids was found [19,20]. Additionally, increased cholesterol levels were observed in fish fed fishmeal-based diets supplemented with taurine [34,37], but also in fish fed taurine-supplemented low-fishmeal diets [12,31,36]. In some studies, the potential hypolipidemic effects of taurine were translated into lower whole-body lipid deposition and/or lower hepatosomatic index [13,14,17,18], but the potential of taurine in accelerating lipogenesis was also described [34]. Thus, dietary taurine supplementation seems to regulate lipid metabolism in multiple ways.

Considering the previous studies, it seems that fish, especially marine carnivorous fish, require a supplemental taurine source when fed plant-protein-based diets to properly modulate physiological functions. Senegalese sole (*Solea senegalensis*) is a marine fish species with high market value and increasing importance for aquaculture production in Southern European countries [38]. Research studies demonstrated tolerance to high dietary inclusion levels of plant-protein sources [39,40,41,42,43], but it was suggested that taurine inclusion in these diets may be beneficial [43]. Furthermore, Senegalese sole has a low tolerance to high levels of dietary lipids [44], and as mentioned before, taurine assumes an important role in lipid digestion and metabolism in fish. Therefore, this study aims to evaluate the effects of taurine supplementation to low-fishmeal diets on the physiological responses of Senegalese sole, with an emphasis on lipid metabolism.

## 2. Materials and Methods

### 2.1. Experimental Diets

Two basal isonitrogenous (~55% crude protein) and isolipidic (8.6% crude fat) diets were formulated (Table 1): a fishmeal-based diet (FM), similar to a commercial diet, and a plant-protein-based diet (PP0), in which plant-protein sources replaced 85% of marine ingredients, as previously used by Aragão [45] and Richard [46]. FM and PP diets contained 0.4 and 0.08% of taurine, respectively (Table 1). Based on the PP0 formulation, two additional diets were further supplemented with microencapsulated taurine (as previously described in Aragão [45]) to obtain dietary taurine levels similar to the FM diet and to the level found in polychaetes, the natural food of Senegalese sole in the wild [47]: diets PP0.5 and PP1.5, respectively. The detailed formulations are depicted in Table 1. All diets were formulated to fulfil the known nutritional requirements (indispensable amino acids and phosphorus) of juvenile Senegalese sole.

All diets were manufactured and extruded at SPAROS Lda. (Olhão, Portugal). Briefly, powdered ingredients were mixed in a double-helix mixer, and diets were extruded (twin-screw extruder, model BC45 (Clextral, Firminy, France)) and dried in a convection oven (OP 750-UF, LTE Scientific, Oldham, UK). After cooling, the oils were added to the pellets by vacuum coating (model PG-10VCLAB (Dinnisen, Sevenum, The Netherlands)). Samples from all diets were collected and analysed for proximate composition, taurine content (Table 1), and amino acid (Table 2) profile, as described in Section 2.4. Throughout the duration of the trial, experimental diets were stored at room temperature in a cool and aerated storage room.

### 2.2. Rearing Trial

Senegalese sole juveniles were acquired from a commercial aquaculture and transported to the Centre of Marine Sciences (CCMAR) facilities (Faro, Portugal). Fish were acclimated to the new rearing conditions (recirculated aquaculture system with flat-bottomed tanks equipped with a mechanical filter, a submerged and a trickling biological filter, a protein skimmer, and a UV steriliser) for three weeks, during which they were fed with the FM diet.

After the three weeks of acclimation, fish were individually weighed under light anaesthesia (300 µL L^−1^ 2-phenoxyethanol (Sigma, Madrid, Spain)), and 35 fish (initial body weight: 13.7 ± 5.0 g) were distributed per tank (area: 0.18 m^2^; volume: 18 L; initial fish density: 2.7 kg m^−2^). A short rearing trial was performed to analyse the impacts of taurine supplementation to plant-based diets on the metabolism of taurine, bile acids, and lipids. For this, fish were kept for six weeks under controlled abiotic conditions (temperature: 19.0 ± 1.1 °C; salinity: 31.7 ± 1.7 ‰; dissolved oxygen: 94.3 ± 5.8% of saturation; ammonia < 0.1 mg L^−1^; nitrites < 0.25 mg L^−1^; photoperiod 12 h L:12 h D). Each diet was randomly assigned to triplicate tanks, and fish were fed at 1.0–1.5% body weight with automatic feeders 12 times per day (from 10h00 to 22h00). The feeding ration was adjusted daily based on the fish feed intake. Water-quality parameters and fish mortality were monitored daily.

At the end of the six weeks, twelve fish per tank were sampled after being deprived of feed for 24 h. All fish were anaesthetised (500 µL L^−1^ 2-phenoxyethanol) and individually weighed. From three fish, blood was collected from the caudal vein using heparinised syringes. Plasma samples were snap-frozen in liquid nitrogen and kept at −20 °C until triglycerides and total cholesterol analyses. These fish were then euthanised (1500 µL L^−1^ 2-phenoxyethanol), and the liver was collected and rapidly transferred to RNAlater (Sigma, Madrid, Spain). Samples were maintained at 4 °C for 24 h and then kept at −80 °C until gene expression analysis. Six fish were euthanised and used for the analysis of somatic indexes. The gallbladders were sampled, snap-frozen in liquid nitrogen, and kept at −80 °C for bile-acid-content analysis. Three fish were used for proximate composition and taurine content, and samples were kept at −20 °C until analysis. The liver of the other three fish were sampled and fixed with 10% buffered formaldehyde at pH 7.2 for histological analysis.

All animal manipulations were carried out in compliance with the European (Directive 2010/63/EU) and Portuguese (Decreto-Lei no. 113/2013 de 7 de Agosto) legislation for the use of laboratory animals and were performed by trained scientists under Group-C licenses from the Direção-Geral de Alimentação e Veterinária, Portugal.

### 2.3. Metabolic Trial

At the end of the six weeks, fish from the same treatment were transferred to one tank and kept for two weeks under the same feeding regime and rearing conditions. Fish were deprived of feed for 24 h, and six fish from each treatment were randomly chosen and transferred to the metabolic flux laboratory at CCMAR. Metabolic trials using radiolabelled triolein were performed to better understand the effects of dietary taurine supplementation on Senegalese sole lipid metabolism. 

The metabolic trials used the in vivo method of tube-feeding described in detail previously [46,48,49,50]. Fish were anaesthetised (300 µL L^−1^ 2-phenoxyethanol) and tube-fed at 0.5% body weight with the experimental diets previously labelled with ^14^C-triolein ([U-^14^C]-triolein,1.85 MBq, Perkin Elmer, Waltham, MA, USA). After tube feeding, fish were allowed to recover from anaesthesia in clean seawater with aeration and then were transferred into individual incubation chambers containing 2 L of clean seawater. Each chamber was hermetically sealed, provided with a gentle oxygen flow, and connected to CO_2_ traps (containing 0.5 M potassium hydroxide) to collect ^14^CO_2_ produced by the fish from the catabolism of ^14^C-triolein. After 24 h of incubation, oxygen flow was stopped, and fish were euthanised (1000 mg L^−1^ MS-222 buffered with sodium bicarbonate, Sigma, Madrid, Spain) inside the chambers. Fish were then taken for sampling. After fish removal, incubation chambers were immediately resealed, and acidification of incubation water was conducted gradually, resulting in the diffusion of any remaining ^14^CO_2_ from the water into the CO_2_ traps. Samples from the incubation chambers (considered to contain ^14^C resulting from fish evacuation) and from the CO_2_ traps were collected for radioactivity counting. Fish liver and viscera were carefully sampled, and the rest of the fish was considered to be the fish body.

Fish samples were completely dissolved in Solvable^TM^ (Perkin Elmer, Waltham, MA, USA) at 50 °C for 24 h. A scintillation cocktail (Ultima Gold XR^TM^; Perkin Elmer, Waltham, MA, USA) was added to all samples (water, traps, and fish tissues), and disintegrations per minute (DPM) were determined in a TriCarb 2910TR low-activity Liquid Scintillation Analyser (Perkin Elmer, Waltham, MA, USA). All samples were corrected for quench and lumex. All the results were expressed as a percentage of the sum of the total DPM [51].

### 2.4. Chemical and Biochemical Analysis

Fish bodies, liver, and viscera from each tank were pooled together (*n* = 3 per treatment), and all samples (diets and fish tissues) were ground before analysis. The contents of moisture (105 °C for 24 h) and ash (combustion at 550 °C for 12 h) were determined in diets and fish bodies. Crude protein (N × 6.25) content was determined in freeze-dried-diet and fish-body samples using an elemental analyser (Elementar Vario EL III, Elementar, Stockport, UK). Crude fat was analysed by petroleum ether extraction using a Soxtherm Multistat/SX PC (Gerhardt, Königswinte, Germany) on freeze-dried-diet, fish-body, liver, and viscera samples.

Freeze-dried samples from experimental diets were analysed for total amino acid content after 48 h acid hydrolysis. Taurine content was analysed in freeze-dried-diet, fish-body, and liver samples. Amino acid and taurine analyses were performed according to the procedures described in Aragão [52].

Bile was collected from gallbladders using sterilised syringes. Due to the very low volumes of bile available, the bile from three fish per tank was pooled before analysis (*n* = 3 per treatment). Total bile-acid content in bile was analysed after acid hydrolysis (6 M HCl for 2 h) using a commercial kit (Trinity Biotech, Co. Wicklow, Ireland). Plasma triglycerides and cholesterol concentrations were analysed using commercial kits (Spinreact, St. Esteve de Bas, Spain) in individual fish plasma samples.

### 2.5. Histological Analyses

The fixed-liver samples were embedded in paraffin, and sections of 5 μm were obtained in a microtome Leica© RM-2155 (Leica, Vienna, Austria) and stained with hematoxylin-eosin for general histological observations. Mounted slides were scanned with a Hamamatsu NanoZoomer C13140-01 (Hamamatsu, Hamamatsu City, Japan), and images were visualised with the NDP.view 2 (Hamamatsu, Japan). Based on Ribeiro [53], liver-tissue integrity and hepatocyte vacuolisation (representing glycogen deposits and/or fat storage normally dissolved during the routine histological process) were compared among treatments.

### 2.6. Gene Expression

#### 2.6.1. RNA Extraction and cDNA Synthesis

Total RNA from the fish liver was extracted using Tri reagent (Sigma, Madrid, Spain) following the manufacturer’s specifications. RNA was purified using the High Pure RNA Isolation Kit (Roche, Basel, Switzerland) and treated with DNase I to avoid genomic DNA amplification during real-time PCR. Total RNA was quantified based on absorbance at 260 nm with a NanoDrop 1000 Spectrophotometer (Thermo Fisher Scientific, Waltham, MA, USA), and integrity was assessed by agarose gel electrophoresis. cDNA was synthesised from 500 ng of purified RNA using the M-MLV Reverse Transcriptase Kit (Invitrogen). Negative control reactions were run without the enzyme. For each sample, reverse transcription was performed in duplicate.

#### 2.6.2. Real-Time PCR (RT-PCR)

Quantification of gene expression by RT-PCR was performed using the StepOnePlus^TM^ Real-Time PCR system (Applied Biosystems, Waltham, MA, USA) with SsoFast EvaGreen chemistry (Bio-Rad, Hercules, CA, USA). Transcriptomic analysis was focused on the expression of target genes related to bile-acid and lipid metabolism. The transcripts analysed were: cytochrome P450 family 7 subfamily A member 1 (*cyp7a1*), solute carrier family 6 member 6 (*taut*), ATP-binding cassette subfamily C member 2 (*abcc2*), and ATP-binding cassette subfamily B member 11 (*abcb11*) for bile-acid metabolism; apolipoprotein A1 (*apoa1*), A4 (*apoa4*), and B (*apob100*), microsomal triacylglycerol transfer protein (*mtp*), very-low-density lipoprotein receptor (*vldlr*), and perilipin 2 (*plin2*) for lipid metabolism. Primers’ sequences for glyceraldehyde-3-phosphate dehydrogenase (*gapdh*) were taken from Infante [54], the ones for *taut* from Pinto [55], and the ones for *apoa1*, *mtp,* and *vldlr* from Borges [56]. The remaining primers were defined with the Beacon designer 7.9 software, and forward primers were designed so that they overlap with an intron. Information on specific primers (GenBank accession numbers, forward and reverse primer sequences, and annealing temperatures) is described in Table 3.

The efficiency of the PCR reaction assay for each gene was previously evaluated to assure it was close to 100%. For the *gapdh*, *abcc2*, *vldlr,* and *plin2* genes, five-point standard curves of a 5-fold dilution series (1:5–1:3125) of pooled cDNA were used for PCR efficiency calculation. In the case of the *cyp7a1*, *taut*, *abcb11*, *apoa1*, *apoa4*, *apob100,* and *mtp* genes, PCR efficiency was calculated using five-point standard curves of a 2-fold dilution series (1:10–1:160) of pooled cDNA. Minus reverse transcriptase controls were checked for every gene. Thermal cycling was initiated with the incubation at 95 °C for 30 s for hot-start polymerase activation. Forty-five cycles of PCR were performed, each consisting of heating at 95 °C for 5 s for denaturing and at a specific temperature depending on the primer pair used (see Table 3) for 10 s for annealing and extension. The specificity of the reactions was assessed by analysis of the melting curves with ramping rates of 0.5 °C every 10 s over a temperature range of 55 to 95 °C. Real-time PCR was performed in duplicates for each gene, and negative controls were run for each reaction. Ct values were determined using the baseline subtracted curve fit method using the StepOnePlus^TM^ Real-Time PCR System Software (Applied Biosystems, Waltham, MA, USA) with a fluorescence threshold automatically set. Relative quantification of the target genes’ transcripts was made following the ∆∆Ct method [57]. *Gadph* was tested for gene expression stability using RefFinder [58], and it was used as a housekeeping gene in the normalisation procedure. The mRNA expression of the target genes was compared among dietary treatments in reference to the expression level of *gadph* in fish fed the FM diet, which was arbitrarily assigned a value of 1. All data are presented after log2 transformation.

### 2.7. Statistical and Data Analysis

Data from the rearing trial were used to calculate key performance indicators—weight gain (WG, 1), daily voluntary feed intake (VFI, 2), feed conversion ratio (FCR, 3), and hepatosomatic (HSI, 4) and viscerosomatic (VSI, 5) indexes:

WG (%) = 100 × (final fish weight − initial fish weight) × initial fish weight^−1^ (1)

VFI (% day^−1^) = 100 × apparent feed intake × ((initial fish weight + final fish weight)/2)^−1^ × days^−1^ (2)

FCR = apparent feed intake × (final fish weight − initial fish weight)^−1^ (3)

HSI (%) = 100 × liver weight × fish weight^−1^ (4)

VSI (%) = 100 × viscera weight × fish weight^−1^ (5)

All data were expressed as means with standard deviation (SD). Before statistical analysis, data expressed as a percentage were transformed (arcsin square root [59]), and all data were checked for normality and homogeneity of variances. Significant differences among groups were assessed by one-way ANOVA when data fulfilled the assumptions for analysis. A Kruskal–Wallis test was performed if data failed the assumptions of normality and homogeneity of variances. When statistically significant variations were found (*p* < 0.05), pairwise comparisons of means were performed with Tukey HSD tests or with Bonferroni corrections, respectively. The control group was set as the FM treatment for the relative quantification of gene expression. Regression analyses between the content of taurine in diets and the fish liver, body, or bile-acid concentration were performed using a linear model. All statistical analyses were performed in IBM SPSS Statistics version 27 (Armonk, NY, USA).

Gene expression data were subjected to a principal component analysis (PCA) to verify differences between fish fed the distinct diet formulations and find potential clusters of observations. The standard *prcomp* R function in the auto-scaled matrices was used for PCA, and score plots were produced for the two first principal components (PC1 and PC2) using the *ggbiplot* and *factoextra* packages for R. Confidence ellipses were included, representing 95% confidence intervals around the centroid value of each data cluster. The function *fviz_cos2* was used to visualise the quality of representation (cos2) of the variables in the PCs. PCA analyses were carried out using the open-source software R version 4.2.1 (R Core team, Vienna, Austria).

## 3. Results

Diet analysis revealed that taurine content (Table 1) in the PP0 diet (0.08%) was five-times lower than in the FM diet (0.40%). Taurine contents were similar in FM and PP0.5 (0.43%) diets, while the PP1.5 diet (1.40%) presented 3.5-times more taurine than the FM diet.

At the end of the short-term rearing trial, fish fed the FM diet doubled their initial weight (Table 4). The ANOVA results indicate that the final weight of fish presents significant differences among dietary treatments (*p* = 0.046); however, post hoc tests were unable to identify those differences. Furthermore, the weight gain of fish fed the PP0 and the PP0.5 diets was significantly inferior (*p* = 0.002) to that of fish fed the FM diet. However, when fish were fed the PP1.5 diet, the weight gain was significantly higher than that of fish from the PP0 and PP0.5 treatments and not significantly different from fish fed the FM diet. Voluntary feed intake, FCR, and survival were similar among the dietary treatments (*p* > 0.05; Table 4).

**Figure 1:**
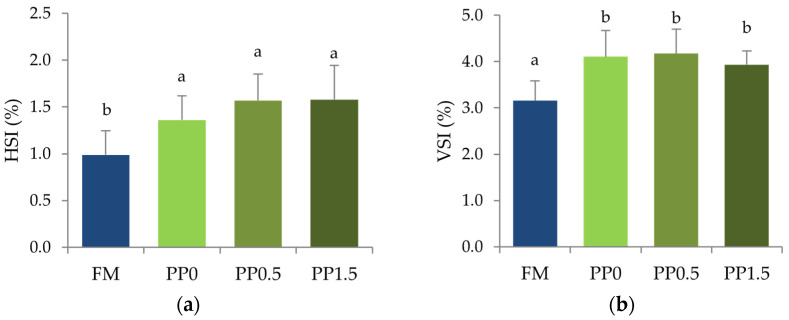
Hepatosomatic (**a—HSI**) and viscerosomatic (**b—VSI**) indexes of Senegalese sole juveniles fed a fishmeal (FM), a plant-based (PP0), or a PP diet supplemented with 0.5% (PP0.5) or 1.5% (PP1.5) of taurine. Values are means + standard deviation (*n* = 6). Different letters indicate significant differences (*p* < 0.05) among dietary treatments.

Fish somatic indexes (Figure 1) were significantly affected by the dietary protein source. In fish fed plant-based diets, both HSI (Figure 1a, *p* < 0.001) and VSI (Figure 1b, *p* < 0.001) were significantly higher than in fish fed the FM diet, irrespective of taurine supplementation.

**Figure 2:**
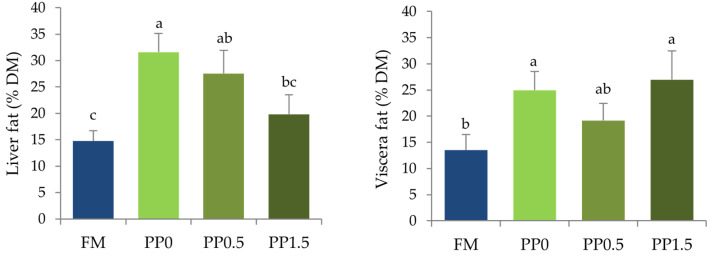
Fat content in the liver (**a**) and viscera (**b**) of Senegalese sole juveniles fed a fishmeal (FM), a plant-based (PP0), or a PP diet supplemented with 0.5% (PP0.5) or 1.5% (PP1.5) of taurine. Values are means + standard deviation (*n* = 3). Different letters indicate significant differences (*p* < 0.05) among dietary treatments. DM = dry matter.

**Figure 3:**
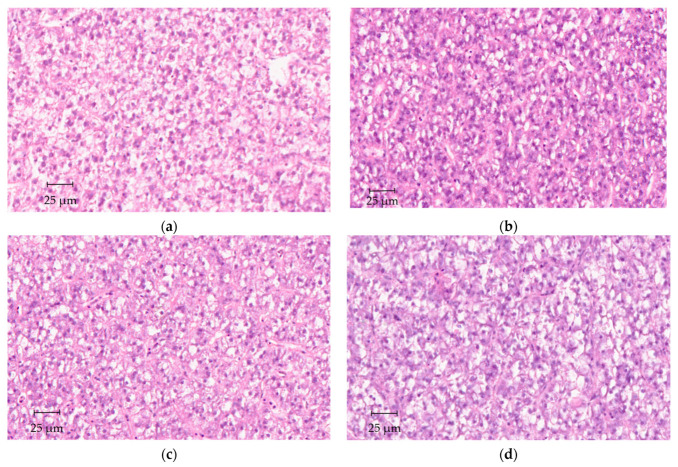
Histological sections (H&E staining) of the liver of Senegalese sole juveniles fed a fishmeal (**FM—a**), a plant-based (**PP—b**), or a PP diet supplemented with 0.5% (**PP0.5—c**) or 1.5% (**PP1.5—d**) of taurine.

Ash content in fish bodies (Table 5) significantly increased in fish fed the PP0 diet compared with FM-fed fish (*p* = 0.014), while intermediate values were found for fish fed the taurine-supplemented diets (PP0.5 and PP1.5). No significant differences (*p* > 0.05) in dry matter and protein contents were observed among dietary treatments. Fat content in fish bodies decreased significantly (*p* < 0.001) in fish fed all the plant-based diets (PP0, PP0.5, PP1.5) compared with FM-fed fish (Table 5). A significant increase (*p* < 0.001) in hepatic fat content was observed in fish fed the PP0 diet compared with FM-fed fish (Figure 2a). Dietary taurine supplementation decreased hepatic fat content, leading to results not significantly different between fish fed the PP1.5 and the FM diets (Figure 2a). As for viscera, an increase in fat content was observed in fish fed the plant-based diets (*p* = 0.009), although results were not significantly different between fish fed the PP0.5 and the FM diets (Figure 2b). Histological analysis revealed no significant differences that could be attributed to diet (Figure 3). Hepatocytes exhibited a polygonal-like shape, disposed along sinusoids in a manner that was similar among dietary treatments. The hepatocyte nucleus was displaced laterally among dietary treatments because of the storage area (vacuolisation-like area), but the variability observed within each treatment was higher than among treatments.

**Figure 4:**
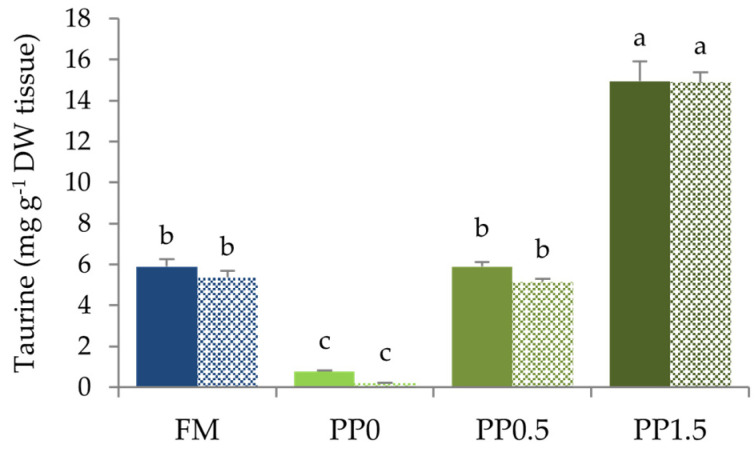
Taurine content in the liver (filled bars) and body (shaded bars) of Senegalese sole juveniles fed a fishmeal (FM), a plant-based (PP0), or a PP diet supplemented with 0.5% (PP0.5) or 1.5% (PP1.5) of taurine. Values are means + standard deviation (*n* = 3). Different letters indicate significant differences (*p* < 0.05) among dietary treatments. DM = dry matter.

**Figure 5:**
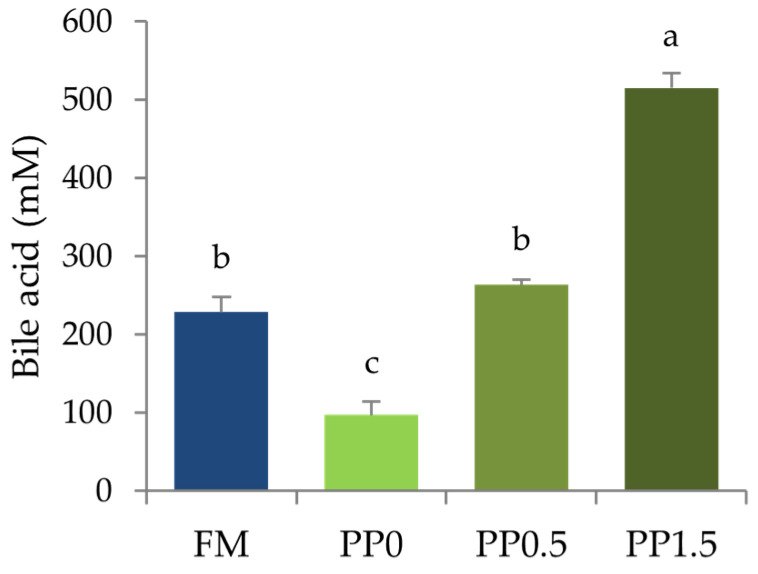
Bile-acid concentration in the bile of Senegalese sole juveniles fed a fishmeal (FM), a plant-based (PP0), or a PP diet supplemented with 0.5% (PP0.5) or 1.5% (PP1.5) of taurine. Values are means + standard deviation (*n* = 3). Different letters indicate significant differences (*p* < 0.05) among dietary treatments.

Feeding fish with the PP diet significantly decreased (*p* < 0.05) taurine contents in fish liver and body (Figure 4). Dietary taurine supplementation linearly increased taurine contents in both fish liver and body, as confirmed by regression analysis (*p* < 0.001, R^2^ = 0.975 and 0.984, respectively). The same pattern was found for bile-acid concentration in fish bile (Figure 5), and this increased linearly with the dietary taurine concentration, as confirmed by regression analysis (*p* < 0.001; R^2^ = 0.973).

**Figure 6:**
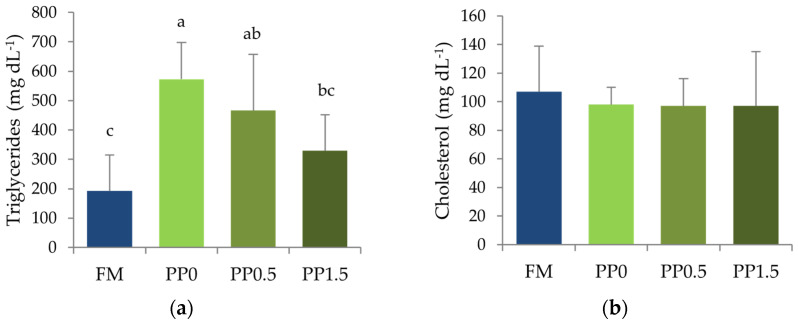
Triglycerides (**a**) and total cholesterol (**b**) concentration in plasma of Senegalese sole juveniles fed a fishmeal (FM), a plant-based (PP), or a PP diet supplemented with 0.5% (PP0.5) or 1.5% (PP1.5) of taurine. Values are means + standard deviation (*n* = 9). Different letters indicate significant differences (*p* < 0.05) among dietary treatments. The absence of letters indicates no significant differences.

Triglycerides levels in plasma were significantly higher (*p* = 0.004) in fish fed the PP0 diet compared with FM-fed fish (Figure 6a). A decrease in plasma triglyceride concentration with an increase in dietary taurine supplementation was observed, thus resulting in no significant differences between fish fed the PP1.5 and the FM diets (Figure 6a). Total cholesterol levels in plasma were not significantly affected (*p* = 0.853) by the dietary treatments (Figure 6b).

**Figure 7:**
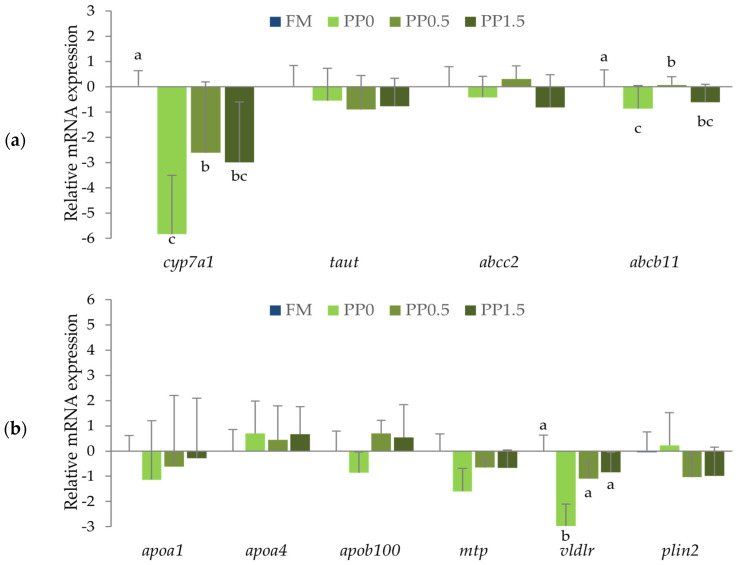
Relative mRNA expression of genes involved in bile-acid metabolism (**a**): *cyp7a1* (cytochrome P450 family 7 subfamily A member 1), *taut* (solute carrier family 6 member 6), *abcc2* (ATP-binding cassette subfamily C member 2), and *abcb11* (ATP-binding cassette subfamily B member 11); or in lipid metabolism (**b**): *apoa1* (apolipoprotein A1), *apoa4* (apolipoprotein A4), *apob100* (apolipoprotein B100), *mtp* (microsomal triacylglycerol transfer protein), *vldlr* (very-low-density lipoprotein receptor), and *plin2* (perilipin 2) of Senegalese sole juveniles fed a fishmeal (FM), a plant-based (PP0), or a PP diet supplemented with 0.5% (PP0.5) or 1.5% (PP1.5) of taurine. Values are means + standard deviation (*n* = 7–9). Different letters indicate significant differences (*p* < 0.05) among dietary treatments. The absence of letters indicates no significant differences.

Fish from the PP0 treatment displayed a downregulation of genes involved in bile-acid metabolism, such as *cyp7a1* and *abcb11*, compared to FM-fed fish (Figure 7a). The mRNA expression levels of these genes significantly increased (*p* > 0.05) in fish fed the PP0.5 diet when compared with animals from the PP0 treatment but were still significantly different from the levels found in the FM treatment. At the highest dose of taurine tested (PP1.5), the expression of *cyp7a1* and *abcb11* presented values not significantly different from those found either in fish fed the PP0 or the PP0.5 diet. Concerning the genes involved in lipid metabolism (Figure 7b), a downregulation of *vldlr* was found in fish fed the PP0 diet compared with the FM-fed fish (*p* = 0.001). mRNA expression levels of *vldlr* significantly increased in fish fed the PP diets supplemented with taurine compared with nonsupplemented ones, and this result was not significantly different from the FM treatment. The mRNA expression levels of the other genes analysed were not significantly different (*p* > 0.05) among treatments.

**Figure 8:**
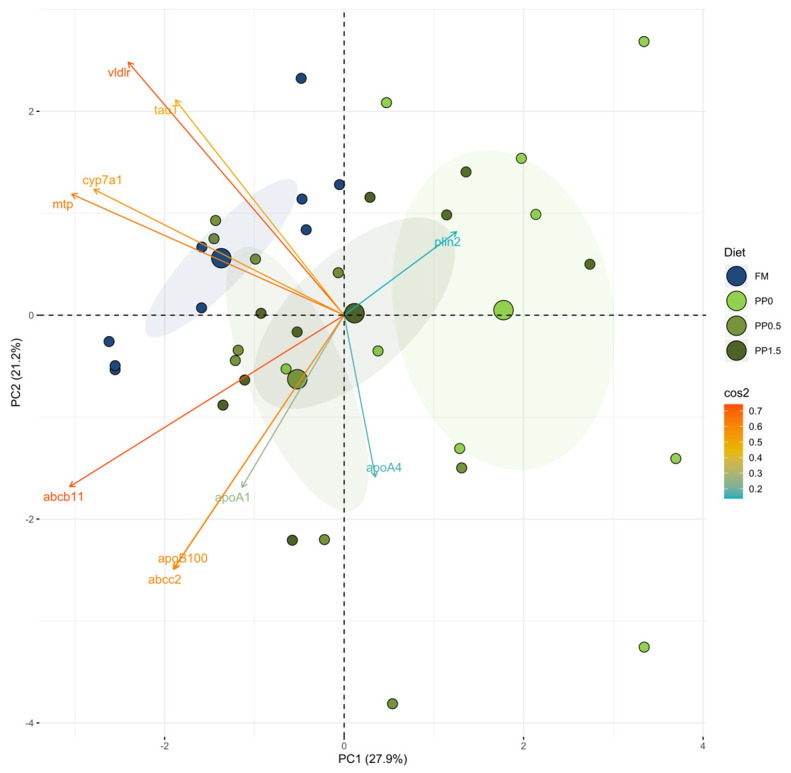
Principal component analysis (PCA) of the relative mRNA expression data in the liver of Senegalese sole juveniles fed the different experimental diets (FM, PP, PP0.5, and PP1.5). Each point represents the projection of an individual sample in the PC1 and PC2 axes. The ellipses represent 95% confidence intervals around the centroid (larger point) of each data cluster.

Principal component analysis (PCA) was used to reduce the complexity of the data from gene expression analysis (Figure 8). Relative mRNA expression of the target genes allowed the differentiation of FM- and PP0-fed fish as two distinct clusters along the PC1 axis. Conversely, it was observed that in the taurine-supplemented treatments (PP0.5 and PP1.5), the clusters were not so distinct and intermingled among the others. The differential expression of the *vldlr* and *abcb11* genes were the most responsible for the obtained dissimilarities, as observed by the darker colour in the cos2 scale.

**Figure 9:**
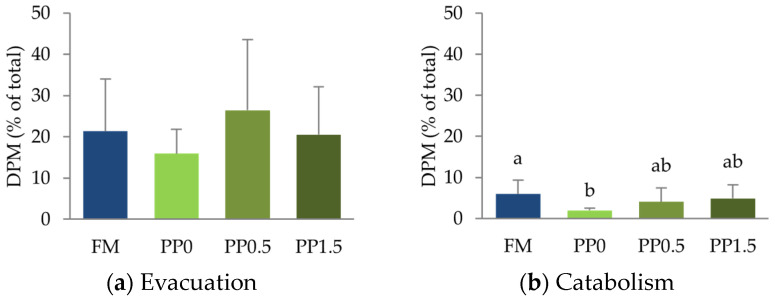

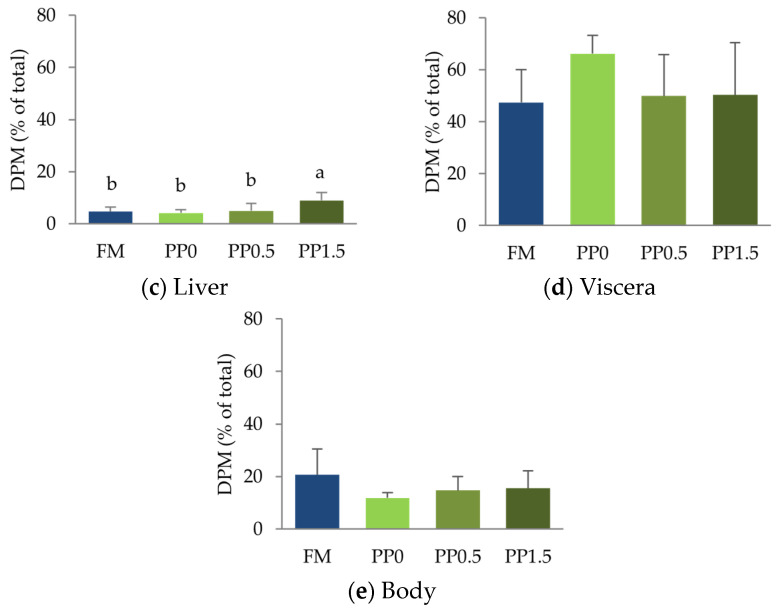
^14^C-Triolein evacuation (**a**), catabolism (**b**), and retention in the liver (**c**), viscera (**d**), and body (**e**) of Senegalese sole juveniles fed a fishmeal (FM), a plant-based (PP0), or a PP diet supplemented with 0.5% (PP0.5) or 1.5% (PP1.5) of taurine. Values are means + standard deviation (*n* = 6). Different letters indicate significant differences (*p* < 0.05) among dietary treatments. The absence of letters indicates no significant differences. DPM = disintegrations per minute.

The results from the metabolic trials showed that a significant part (21 ± 12%) of the ^14^C-triolein was not absorbed by the Senegalese sole and was evacuated (Figure 9a) without a significant impact of the dietary treatments (*p* = 0.757). Catabolism of ^14^C-triolein was significantly reduced (*p* = 0.045) in fish fed the PP0 diet compared with FM-fed fish (Figure 9b), while taurine-supplemented treatments presented intermediate values. Hepatic ^14^C-triolein retention was significantly higher (*p* = 0.011) in the PP1.5 treatment compared with the other treatments (Figure 9c). The retention of ^14^C-triolein in fish viscera (Figure 9d) and body (Figure 9e) was not significantly affected (*p* > 0.05) by the dietary treatments, and the results indicate that the major proportion (53 ± 16%) of ^14^C-triolein was retained in the fish viscera (Figure 9d).

## 4. Discussion

In contemporary aquafeed formulations, the amounts of fishmeal are being constantly reduced, and the inclusion of plant-protein sources is now a standard procedure. Given the important physiological role of taurine and its minimal content in high-plant diets, the importance of testing the effects of its dietary supplementation for marine carnivorous fish seems fundamental. The current study was a short-term feeding trial to analyse the effects of taurine supplementation to plant-based diets on the metabolism and physiological responses of Senegalese sole. 

Dietary taurine levels analysed went according to what was expected, as a clear reduction in taurine levels was found in the PP0 diet compared with the FM diet. However, by reducing the dietary fishmeal and increasing the plant-protein sources, some changes in the dietary amino acid profile were observed. Since taurine is the end product of sulphur amino acid metabolism, dietary methionine and cysteine levels may present interactive effects with taurine. However, it should be emphasised that the diets were formulated to fulfil the known indispensable amino acid requirements of the species. Published works indicate no interaction between dietary taurine and methionine levels on meagre performance and metabolism [36,60], while a possible sparing effect of taurine on methionine requirements has also been described [16,61]. Though taurine is virtually absent in terrestrial plants, the residual content found in the PP diet is the result of the inclusion of marine ingredients known to be rich in taurine, such as fishmeal, fish soluble protein concentrate, and even squid meal [62]. Taurine supplementation to plant-based diets resulted as planned, since the PP0.5 diet presented taurine levels similar to the FM diet, while in the PP1.5 diet, taurine content was within the range found in polychaetes [63], which Senegalese sole feed on in the wild [47]. The above-mentioned marine ingredients were included at low levels in the plant-based diets to act as feed attractants and ensure good diet acceptability. This objective was attained since no significant differences were found in voluntary feed intake among treatments. Although taurine is considered a feeding attractant, the low inclusion levels of marine ingredients in the plant diets may explain the absence of effects of taurine supplementation on fish-feed intake, contrary to other studies that reported its increase in fish fed plant-based diets supplemented with taurine [15].

Even though the short duration of the trial does not allow for robust considerations on the effects of dietary taurine supplementation on growth performance, these results seem pertinent, as it is possible to verify that the fish were in good rearing conditions and doubled their weight when fed the FM diet for 6 weeks. Taking into consideration the limitations previously acknowledged, it is possible to describe a negative effect of the high dietary replacement of fishmeal by plant-protein sources on the growth performance of Senegalese sole. This negative effect should be analysed with caution due to the short-term nature of the trial since previous studies have shown a good acceptance of high-plant diets [39,40,41,42,43]. It should be noted that in this study, 85% of marine ingredients were replaced by plant ingredients, resulting in a low inclusion level of the former (9% of fishmeal, fish soluble protein concentrate, and squid meal) compared with the previous studies (min. 15% of marine ingredients). The negative effects of the PP0 diet on growth performance were at least partially mitigated through taurine supplementation. In similar short-term trials with sturgeon, taurine inclusion in high-plant diets increased growth performance, but only at low levels, resulting in negative effects on growth performance when included at supraoptimal levels [30,31]. Interestingly, with Senegalese sole, growth performance in the PP0.5 treatment presented intermediary values between fish fed the FM and the PP0 diets, and only at the highest dietary taurine supplementation (PP1.5) was the fish growth performance similar to the FM treatment. This does not seem to be related to increasing feed palatability, as mentioned before, since no effects of taurine supplementation were observed in the fish voluntary feed intake. Previous studies with Japanese flounder (*Paralichthys olivaceus*) and turbot (*Scophthalmus maximus*) showed that the optimal dietary taurine level decreased as fish grew [64,65]. This suggests that in high-plant diets for young Senegalese sole, taurine supplementation should be above the levels found in commercial fishmeal-based diets.

The improved fish growth performance as a result of dietary taurine supplementation is probably the reflection of the several effects observed on fish physiology and metabolism. The dietary taurine content was strongly correlated with the taurine levels in tissues (liver and body), as previously observed in other fish species, such as Japanese flounder, turbot, cobia (*Rachycentron canadum*), white grouper (*Epinephelus aeneus*), red seabream, Nile tilapia, and African catfish, irrespectively of the main dietary protein source [12,16,24,25,37,64,65]. The increase in taurine levels in the fish muscle (which comprises the major portion of the fish body) may represent a potential health benefit for consumers, as suggested by Watson [37], providing an additional source of a nutrient known for its hypolipidemic and antiatherogenic effects in humans [33].

The different hepatic taurine contents had no effect on the HSI, as previously observed in other fish species fed low fishmeal diets [8,25,27,60,66]. Additionally, the somatic indexes were only affected by the dietary protein source, similar to other works that found an increased HSI and VSI in Senegalese sole fed high-plant diets [42,43]. No histological alterations in hepatocytes were observed in the current study, contrary to the increased hepatic vacuolisation and necrosis found previously in Senegalese sole fed high-plant diets [43]. This might be related to the low dietary fat levels used in this study (~8%), since previous works indicated moderate hepatic steatosis and cellular necrosis when Senegalese sole were fed fishmeal-based diets with 15% of crude fat, but not with 8% [43]. The dietary protein source also affected fat deposition in fish tissues. As in other studies with Senegalese sole fed high-plant diets [42] and also with black carp [13], body fat levels were lower in PP-fed fish compared with FM-fed fish, and no effects of taurine were observed. Senegalese sole is a lean fish, presenting low fat content in muscle, with the liver and viscera functioning as important fat storers [44]. The results from the metabolic trial corroborate that triolein retention occurred mostly in the viscera, irrespective of the dietary protein source. Furthermore, lipid content in the liver and viscera increased in fish fed the plant-based diets, which is in line with the observed increase in somatic indexes. Previously, it has been suggested that this increase seems to be due to augmented triglyceride storage [43], and metabolic trials have demonstrated a lower capacity of PP0-fed fish to catabolise triolein, although it was further mitigated by taurine supplementation.

A similar metabolic trial with ^14^C-triolein was previously performed with Senegalese sole juveniles [46]. Interestingly, while in the current study dietary taurine supplementation to plant-based diets increased triolein catabolism, in the previous study, triolein absorption augmented concomitantly with increasing dietary taurine levels, without effects on catabolism or retention. These differences may be related to the timeframe of exposure to the experimental diets, since in the previous study Senegalese sole were fed the experimental diets only for five days and after a period of taurine deprivation. The short-time habituation to the diets resulted in evacuation of almost 60% of ^14^C-triolein in fish fed a high-plant-based diet without taurine supplementation. This probably impaired the further metabolism of triolein, since in the present study triolein evacuation was only 20% for all dietary treatments. These effects of taurine on lipid digestion and metabolism seem to be linked with the impacts on bile-acid metabolism. Bile-acid concentration in Senegalese sole’s bile was linearly related to dietary taurine content. Thus, the replacement of fishmeal by plant proteins led to a significant reduction in bile-acid content, which was also observed in white seabream (*Diplodus sargus*) [20]. However, as in other studies using low-fishmeal diets [20,25,28,36], taurine supplementation to the plant-based diet led to a significant increase in bile-acid content. Bile acids are synthesised from cholesterol and conjugated with taurine to form bile salts, and the rate-limiting enzyme is *cyp7a1* [5], which was downregulated in fish fed the PP diet and significantly increased with taurine supplementation, as previously observed in black seabream (*Acanthopagrus schlegelii*) [26]. As cholesterol circulating levels were not affected by the dietary treatments while taurine concentrations were linearly affected by the dietary taurine content, bile-acid synthesis in the current study seems to be limited by taurine availability. Similar results were observed in white seabream fed low-fishmeal diets, in which the increased taurine supplementation led to a higher concentration of bile salts, with concomitant lower levels of plasma triglyceride but without effects on cholesterol [20]. Although a relation between hepatic triglyceride accumulation and increased fat content seems to be supported by the current results, at this point, a possible hepatic accumulation of cholesterol in fish fed the PP0 diet cannot be excluded, as a result of the downregulation of *cyp7a1* and, to some extent, of *vldlr*. The increase in hepatic cholesterol when lowering fishmeal contents in diets for yellowtail was previously observed, and this effect was reverted by taurine supplementation [67]. 

The expression levels of *abcc2* and especially of *abcb11* (which encode for the transporter proteins that actively transport secreted molecules into bile [6]) followed a similar pattern to that of *cyp7a1*, indicating an increased bile-acid secretion in fish fed PP diets supplemented with taurine in comparison to nonsupplemented ones. Disturbances in bile-acid excretion were previously related to the appearance of green liver [22,23,32]. In this study, hepatic appearance was not affected by dietary treatments, but the possible effects of long-term feeding should not be disregarded, since, as previously mentioned, Senegalese sole reared to the market size with high-plant diets presented histopathological signs [43]. The hepatic *taut* mRNA expression levels were not affected, which is similar to what was observed in cobia [37]. In the latter, despite an increase in taurine contents in the fillet and liver with increasing dietary taurine levels, hepatic *taut* transcript levels did not vary. The authors suggested that the transporter may be upregulated in other tissues to facilitate the recycling of taurine at the lower levels of dietary input, which can also be a possibility in the current study. For instance, it was previously identified that *taut* in the Senegalese sole intestine was more expressed in the hindgut, suggesting an enterohepatic recycling pathway for maintaining taurine levels in the body [55], with implications for bile-salt synthesis. 

The gallbladder bile-acid content was inversely related to hepatic (and to some extent to body) fat accumulation and to the plasma triglycerides levels. In line with this, the metabolic trials showed a decrease in triolein catabolism in the PP treatment, which was partially reverted through taurine supplementation. Surprisingly, hepatic triolein retention was higher in PP1.5 treatment, but this seems to be a transient effect, as the hepatic fat content was significantly lower than in PP0-fed fish. This suggests a better utilisation of triglycerides in the presence of increased levels of taurine and/or bile acids. The effects of taurine on circulating triglycerides levels were not so evident in other fish species, even if comparing only with studies using low-fishmeal diets, with no effects observed in European seabass (*Dicentrarchus labrax*) [28,29], increased plasma levels found in meagre (*Argyrosomus regius*) and African catfish [12,36], but, like in this study, decreased levels found in white seabream, black carp, and yellow catfish [13,14,20]. The expression levels of *vldlr* and up to some level of *mtp* genes indicate a disruption in lipid transport in fish fed the PP diet, which was at least partially reverted by taurine supplementation. The results of the metabolic trials indicate that lipid digestion was not affected by the different bile-acid content, but these seem to lead to better lipid utilisation. The hypolipidemic effects of taurine found in mammals [33] and in some fish species [2] were thus also found in Senegalese sole.

The effects of taurine supplementation to plant-based diets on the mRNA expression levels of the genes involved in bile-acid and lipid metabolism were mostly mild, but the PCA analysis of these data clearly demonstrated a high separation between clusters from fish fed the FM and the PP0 diets. Even more interesting is that the clusters of fish fed taurine-supplemented diets were located in between. Therefore, the increased taurine concentrations in Senegalese sole tissues with a concomitant increase in bile-acid concentrations led to impacts on bile-acid and lipid metabolism that may explain the positive effects of taurine supplementation to plant-based diets. This is in line with the previous discussion indicating that taurine supplementation partially reverted the negative effects of plant-protein-based diets and explains the potential beneficial effects on fish growth performance.

In this study, low-lipid diets were used, since it was previously established that Senegalese sole does not tolerate high dietary lipid levels [44]. Given the positive impact of dietary taurine on lipid metabolism, further studies looking at the effects of dietary taurine supplementation on high-lipid diets for Senegalese sole seem to be worthy of investigation.

## 5. Conclusions

This study showed that dietary inclusion of high levels of plant ingredients to replace fishmeal and other marine ingredients resulted in disturbances in the lipid metabolism of Senegalese sole, due to the resultant low bile-acid concentration and/or the limited availability of taurine for bile-salt emulsification. On the opposite side, increasing levels of taurine in plant-based diets resulted in a higher accumulation of taurine in tissues (liver and body) and in increasing bile-acid concentration. This was associated with an upregulation of *cyp7a1* and *abcb11*, indicating an increase in bile-acid production and secretion, respectively. Thus, taurine supplementation mitigated part of the negative effects of plant-based diets, leading to better lipid utilisation. Additionally, although taurine supplementation in plant-based diets had a positive effect on growth performance, this seems to be necessary at levels that go beyond those found in commercial diets based on fishmeal. These positive effects may eventually have a higher impact on growth performance and physiological condition of Senegalese sole in long-term trials.

## Figures and Tables

**Table 1:** Formulation and analysed proximate composition of experimental diets.

	Diets			
Ingredients	FM	PP0	PP0.5	PP1.5
Fishmeal Super Prime ^1^	37.00	3.00	3.00	3.00
Fishmeal 60 ^2^	15.00	0.00	0.00	0.00
Fish soluble protein concentrate ^3^	7.50	3.00	3.00	3.00
Squid meal ^4^	7.50	3.00	3.00	3.00
Fish gelatine ^5^	2.00	2.00	2.00	2.00
Pea protein concentrate ^6^	0.00	20.00	20.00	20.00
Potato protein concentrate ^7^	0.00	12.00	12.00	12.00
Wheat gluten ^8^	0.00	10.00	9.45	7.70
Corn gluten meal ^9^	0.00	8.00	8.00	8.00
Soybean meal ^10^	5.00	5.00	5.00	5.00
Soy protein concentrate ^11^	5.00	5.00	5.00	5.00
Wheat meal ^12^	6.40	5.10	5.10	5.10
Pea starch ^13^	7.00	7.00	7.00	7.00
Fish oil ^14^	2.50	6.80	6.80	6.80
Vitamin and mineral premix ^15^	0.20	0.20	0.20	0.20
Lutavit C35 ^16^	0.10	0.10	0.10	0.10
Lutavit E50 ^17^	0.05	0.05	0.05	0.05
Monocalcium phosphate ^18^	0.00	4.00	4.00	4.00
Glycerol ^19^	2.50	2.50	2.50	2.50
Binder ^20^	2.00	2.00	2.00	2.00
Antioxidant ^21^	0.25	0.25	0.25	0.25
Lysine ^22,^*	0.00	0.50	0.50	0.50
Methionine ^23,^*	0.00	0.50	0.50	0.50
Taurine ^24,^*	0.00	0.00	0.55	2.30
**Proximate composition (% as fed)**				
Dry matter	92.7	92.4	92.6	92.5
Ash	9.2	9.2	9.3	9.1
Crude protein	55.2	54.9	55.1	55.0
Crude fat	8.6	8.6	8.6	8.6
Taurine	0.40	0.08	0.43	1.40

All values are reported as the mean of duplicate analysis. ^1^ Super Prime: 66.3% crude protein (CP), 11.5% crude fat (CF), Pesquera Diamante, Lima, Peru. ^2^ Fair Average Quality (FAQ) fishmeal: 62% CP, 12% CF, COFACO, Lisboa, Portugal. ^3^ CPSP 90: 84% CP, 12% CF, Sopropêche, Wimille, France. ^4^ Super prime without guts: 84% CP, 4.7% CF, Sopropêche, Wimille, France. ^5^ Fish gelatine: 95% CP, WEISHARDT International, Liptovský Mikuláš, Slovakia. ^6^ NUTRALYS F85F: 83% CP, 1% CF, Roquette Frères, Lestrem, France. ^7^ Potato protein concentrate: 76% CP, 1.3% CF, AgroKorn, Videbæk, Denmark. ^8^ VITAL: 85.7% CP, 1.3% CF, Roquette Frères, Lestrem, France. ^9^ GLUTALYS: 61% CP, 6% CF, Roquette Frères, Lestrem, France. ^10^ Micronised soybean meal: 51% CP, 2.9% CF, SORGAL SA, Aveiro, Portugal. ^11^ Soycomil P: 65% CP, 0.8% CF, ADM, Amsterdam, The Netherlands. ^12^ Wheat meal: 10.2% CP, 1.2% CF, Casa Lanchinha, Alhos Vedros, Portugal. ^13^ NASTAR: 90% starch, COSUCRA, Warcoing, Belgium. ^14^ Marine oil omega 3: Henry Lamotte Oils GmbH, Bremen, Germany. ^15^ PVO40.01 Premix for marine fish, PREMIX Lda, Neiva, Portugal. Vitamins (per kg diet): 100 mg DL-alpha tocopherol acetate, 25 mg sodium menadione bisulphate, 20,000 IU retinyl acetate, 2000 IU DL-cholecalciferol, 30 mg thiamine, 30 mg riboflavin, 20 mg pyridoxine, 0.1 mg B12, 200 mg nicotinic acid, 15 mg folic acid, 3 mg biotin, 100 mg calcium pantothenate, 1 g ascorbic acid, 0.5 g inositol, 1 g choline chloride, and 0.5 g betaine. Minerals (per kg diet): 2.5 mg cobalt sulphate, 1.1 mg copper sulphate, 0.2 g ferric citrate, 5 mg potassium iodide, 15 mg manganese sulphate, 0.2 mg sodium selenite, 40 mg zinc sulphate, 0.6 g magnesium hydroxide, 1.1 g potassium chloride, 0.5 g sodium chloride, and 4 g calcium carbonate. ^16^ Lutavit C35, BASF, Ludwigshafen am Rhein, Germany. ^17^ Lutavit E50, BASF, Ludwigshafen am Rhein, Germany. ^18^ MCP: 220 g kg^−1^ P, 180 g kg^−1^ Ca, Beernem Fosfitalia, Ravenna, Italy. ^19^ From rapeseed, BELGOSUC, Beernem, Belgium. ^20^ Kieselguhr, LIGRANA GmbH, Eilsleben, Germany. ^21^ Paramega PX, Kemin Europe NV, Herentals, Belgium. ^22^ L-Lysine HCl 99%: Ajinomoto Eurolysine SAS, Paris, France. ^23^ DL-Methionine 99%: EVONIK Nutrition & Care GmbH, Krefeld, Germany. ^24^ L-Taurine 98.5%: Ajinomoto Eurolysine SAS, Paris, France. * Supplemented amino acids were microencapsulated in gelatine, according to Aragão [45].

**Table 2:** Analysed amino acid composition of experimental diets.

Amino Acids	Diets			
(% as Fed)	FM	PP0	PP0.5	PP1.5
Arginine	3.9	3.6	3.7	3.7
Histidine	1.4	1.1	1.2	1.2
Lysine	4.2	4.1	4.1	4.1
Threonine	2.1	1.8	1.9	1.9
Isoleucine	2.3	2.2	2.3	2.3
Leucine	3.5	4.2	4.2	4.2
Valine	2.6	2.6	2.6	2.5
Methionine	1.7	1.3	1.2	1.2
Phenylalanine	2.2	2.6	2.5	2.6
Cystine	0.2	0.3	0.3	0.3
Tyrosine	1.8	1.9	1.9	2.0
Aspartic acid	4.3	4.0	4.1	4.1
Glutamic acid	6.8	10.5	10.4	10.6
Alanine	2.7	2.2	2.2	2.2
Glycine	3.3	2.1	2.1	2.1
Proline	2.3	3.5	3.5	3.5
Serine	2.2	2.5	2.5	2.5

All values are reported as the mean of duplicate analysis.

**Table 3:** Forward and reverse primers for real-time PCR.

Gene	Genbank/Unigene * Accession Number	Forward Primer (5′-3′)	Reverse Primer (5′-3′)	AT ^1^ (°C)
*gapdh*	AB291587	AGCCACCGTGTCGCCGACCT	AAAAGAGGAGATGGTGGGGGGTGGT	64
*cyp7a1*	416791 *	GCCTACAGTGCCAGAGAGAAC	GCGAAGCCCAAAGCAGTG	64
*taut*	HQ148721.1	CCGAAAGCTGTGTCCATGATG	CAATAGAGGTGATCTGTCCTTCCA	63
*abbc2*	XM_044046365.1	GCTTACATCCACGACTGCTTCCAA	ACATCCTGACTGACGCCTTCCTT	60
*abcb11*	29210 *	AAGCAGAACAACCAGCCATCAGG	CCACCACCATCATCAGCACATCTT	60
*apoa1*	FF283994	TTGAGGCTAATCGTGCCAAA	CCTGCGTGCTTGTCCTTGTA	62
*apoa4*	KP842775.1	AGGAACTCCAGCAGAACCTG	CCTGCGTGCTTGTCCTTGTA	60
*apob100*	14427 *	CCGCTGAGATGGAGAGATA	CTGGGTCATCTTGGAGAAGG	64
*mtp*	KC888960	TGGCACGTTACTGTGGACAT	CCAGGGCAGAGATGATTC	63
*vldlr*	AJ_879619.2	CTGTGTTTGAGGACCGAGTGTT	GACCTGCGTCTTCTTGCTCT	64
*plin2*	185823 *	CTGTCTGGTCCTTGTCTC	GCCTTGCTGAAGTTAGTG	56

*gapdh*: glyceraldehyde-3-phosphate dehydrogenase; *cyp7a1*: cytochrome P450 family 7 subfamily A member 1; *taut*: solute carrier family 6 member 6; *abcc2*: ATP-binding cassette subfamily C member 2; *abcb11*: ATP-binding cassette subfamily B member 11; *apoa1*: apolipoprotein A1; *apoa4*: apolipoprotein A4; *apob100*: apolipoprotein B; *mtp*: microsomal triacylglycerol transfer protein; *vldlr*: very-low-density lipoprotein receptor; *plin2*: perilipin 2. ^1^ AT: annealing temperature.

**Table 4:** Growth, feed utilisation, and survival of Senegalese sole juveniles fed a fishmeal (FM), a plant-based (PP0), or a PP diet supplemented with 0.5% (PP0.5) or 1.5% (PP1.5) of taurine.

Dietary Treatments	FM	PP0	PP0.5	PP1.5
IBW (g) ^1^	13.7 ± 5.0	13.7 ± 5.7	13.6 ± 4.6	13.6 ± 4.9
FBW (g) ^2^	27.6 ± 6.8	24.2 ± 7.0	24.2 ± 5.3	27.1 ± 7.3
WG (%) ^3^	102.6 ± 10.9 ^a^	77.2 ± 4.8 ^b^	77.5 ± 4.5 ^b^	98.9 ± 5.2 ^a^
VFI (% day^−1^) ^4^	1.2 ± 0.01	1.2 ± 0.04	1.2 ± 0.03	1.2 ± 0.01
FCR ^5^	1.7 ± 0.2	2.3 ± 0.4	1.7 ± 0.3	2.0 ± 0.1
Survival (%)	83.8 ± 7.2	85.7 ± 7.6	83.8 ± 3.3	75.2 ± 5.9

Values are means ± standard deviation (*n* = 3, except for fish weight). Different superscripts within the same row indicate significant differences (*p* < 0.05) among dietary treatments. The absence of superscripts indicates no significant differences (except for final body weight, see text). ^1^ Initial body weight (*n* = 35), ^2^ final body weight (*n* = 36), ^3^ weight gain, ^4^ daily voluntary feed intake, ^5^ feed conversion ratio.

**Table 5:** Body proximate composition of Senegalese sole juveniles fed a fishmeal (FM), a plant-based (PP0), or a PP diet supplemented with 0.5% (PP0.5) or 1.5% (PP1.5) of taurine.

Dietary Treatments	FM	PP0	PP0.5	PP1.5
Dry matter (%)	74.8 ± 1.1	75.0 ± 0.4	75.5 ± 0.7	76.0 ± 1.3
Ash (% DM)	7.0 ± 0.2 ^b^	9.3 ± 1.2 ^a^	7.9 ± 0.4 ^ab^	8.2 ± 0.6 ^ab^
Protein (% DM)	67.2 ± 1.4	68.2 ± 0.7	69.3 ± 1.0	69.4 ± 0.7
Fat (% DM)	19.9 ± 1.9 ^a^	16.3 ± 1.6 ^b^	15.3 ± 0.7 ^b^	13.7 ± 1.0 ^b^

Values are means ± standard deviation (*n* = 3). Different superscripts within the same row indicate significant differences (*p* < 0.05) among dietary treatments. The absence of superscripts indicates no significant differences. DM = dry matter.

## Data Availability

The data presented in this study are available on request from the corresponding author.
